# Stabilisation of Recombinant Human Basic Fibroblast Growth Factor (FGF-2) against Stressors Encountered in Medicinal Product Processing and Evaluation

**DOI:** 10.3390/pharmaceutics13111762

**Published:** 2021-10-21

**Authors:** Leah R. Benington, Gunesh Rajan, Cornelia Locher, Lee Yong Lim

**Affiliations:** 1 Division of Pharmacy, School of Allied Health, University of Western Australia, Perth 6009, Australia; leah.benington@uwa.edu.au (L.R.B.); connie.locher@uwa.edu.au (C.L.); 2 Otolaryngology, Head & Neck Surgery, Division of Surgery, Medical School, University of Western Australia, Perth 6009, Australia; gunesh.rajan@luks.ch; 3 Department of Otolaryngology, Head & Neck Surgery, Luzerner Kantonsspital, 6000 Luzern, Switzerland

**Keywords:** fibroblast growth factor 2, basic fibroblast growth factor, stabilisation, thermal stability, processing stability

## Abstract

Basic fibroblast growth factor (FGF-2) is a highly labile protein with strong potential for tissue engineering. The aim of this study was to develop FGF-2 formulations that are stable against physical stressors encountered in pharmaceutical processing and evaluation. Pharmaceutical excipients, alone or in combination, were added to aqueous FGF-2 (770 ng/mL) solution and the stability of the resulting solutions on storage at 4–37 °C was evaluated. Stability of the solutions to repeated freeze-thaw cycles and lyophilisation was also evaluated, as well as the stability of the lyophilised stabilised protein to storage at −4, 4 and 18 °C for up to 12 months. In all of these experiments FGF-2 was quantified by ELISA assay. The as-received FGF-2, when dissolved in water, was highly unstable, retaining only 50% of baseline protein content after 30 min at 37 °C or 1 h at 25 °C. By contrast, FGF-2 solutions prepared with 0.5% *w/v* methylcellulose (MC) and 20 mM alanine (formulation F5) or with 0.5% *w/v* MC and 1 mg/mL human serum albumin (HSA) (formulation F6) were highly stable, having residual FGF-2 content comparable to baseline levels even after 2 h at 37 °C and 5 h at 25 °C. F5 and F6 were also highly stable to repeated freeze-thaw cycles, with >99% of FGF-2 load remaining after the third cycle. In addition, F5 and F6 were stable to lyophilisation, and the lyophilised products could be stored at −4, 4 or 18 °C for at least 12 months, with less than 1% loss in mean FGF-2 content. Thus, FGF-2 solution is effectively stabilised against both thermal and processing stressors in the presence of MC and alanine (F5), or MC and HSA (F6). The resultant FGF-2 solutions may be applied as medicinal products or further processed into more advanced medicinal products, e.g., scaffolds, for wound healing and tissue regeneration.

## 1. Introduction

Basic fibroblast growth factor (FGF-2) is an endogenous, 18-kDa heparin-binding protein that promotes cellular proliferation, migration and differentiation of a variety of tissues [1,2]. Several therapeutic applications for FGF-2 have been identified in the literature [3], with the efficacy of FGF-2 for the treatment of a wide variety of conditions, such as burns [4], mouth ulcers [5], fractures [6], pressure and diabetic ulcers [7,8] and critical limb ischemia [9] evaluated in various clinical trials. One of the largest areas of research relates to the use of FGF-2 for wound healing applications. Chronic wounds often have reduced FGF-2 concentrations, which may account for their poor rates of healing and revascularisation [10,11]. FGF-2 has been extensively evaluated for chronic wound management, and some success has been demonstrated in vitro [12,13,14]. However, the translation of FGF-2 into clinical use has been severely limited by its inherent instability, in particular in aqueous solutions.

FGF-2 is very quickly degraded, making it highly challenging to formulate it into acceptable medicinal products [15,16]. A cluster of positively charged residues located on one side of the FGF-2 structure is thought to constitute the heparin-binding region of the protein, and the latent instability of FGF-2 has been attributed to the significant amount of structural energy associated with this region [17,18,19]. As a result, one stabilisation approach is to add heparin to FGF-2 formulations. This endogenous stabiliser has been shown to protect the FGF-2 against trypsin-, heat-, acid- and protease-mediated inactivation [17,18,19]; however, heparin is not innocuous, and its anticoagulant activity poses significant safety concern as an excipient [17,18,19]. Manufacturers apply lyophilisation and storage at −20 °C to extend the shelf life of formulated FGF-2, however, the formulation does not protect the protein when the lyophilised powder is reconstituted into solutions [17], as the solubilised FGF-2 is highly vulnerable to aggregation and precipitation, resulting in rapid loss of biological function [17]. This is largely due to the presence of two exposed thiol groups on the FGF-2 surface which promote the formation of disulfide-linked multimers, and aggregates [20]. Additionally, storage of the reconstituted FGF-2 solutions at −20 °C is not readily attainable in clinical applications. A comprehensive review of FGF-2 stability and methods for its stabilisation has recently been published [20].

Other alternatives, e.g., to reconstitute lyophilised FGF-2 on demand or to dose FGF-2 more frequently, are achievable only with highly compliant patients. Lou et al. found 31.3% of participants in a clinical trial to be noncompliant, their tendency to administer FGF-2 at higher than prescribed doses raising the risk of serious adverse events [21]. There is therefore strong impetus to develop effective, safe and practical stabilisation strategies to enable FGF-2 aqueous solution to be applied clinically or be further processed into other more sophisticated medicinal products.

Different approaches have been reported for preserving FGF-2 in aqueous solutions [22,23,24]. These range from simple addition of excipients to complex chemical modifications, including genetic re-engineering. The addition of approved pharmaceutical excipients is preferred because this approach is relatively simple, cost-effective and robust to implement. Excipients for stabilising protein solutions include salts, sugars, polymers, proteins and amino acids. Salts (e.g., sodium chloride, sodium nitrate) stabilise the tertiary structure of proteins by shielding undesirable ionic interactions [25,26]. Sugars (e.g., glucose, trehalose) increase the surface tension and viscosity of solutions to minimise protein aggregation [25,26]. Water-soluble polymers (e.g., polyethylene glycol, methylcellulose) also increase solution viscosity, in addition they limit the intra- and inter- bonding between amino acids in the protein [25]. The addition of another protein (e.g., human serum albumin) or amino acids (e.g., alanine, glycine) provides stabilisation through ionic, electrostatic and hydrophobic interactions [26,27].

A customised approach is required for FGF-2 as there is no ‘one size fits all’ strategy to protein stabilisation. To date, a systematic approach to stabiliser selection has not been reported for FGF-2, and most stability studies have been directed at sustaining FGF-2 activity in cell cultures, with a smaller number aiming to develop sustained release FGF-2 formulations for tissue engineering. However, the capacity of stabilised FGF-2 solutions to withstand the stressors of medicinal product manufacture and testing has not been reported, and there is as yet no published data on long term storage stability, a critical predictor of the commercial viability of a medicinal product formulation.

The aims of this study were to apply a systematic approach to identify and optimise stabilisation vehicles for FGF-2, and to evaluate the stability of the final stabilised FGF-2 formulations against thermal and processing stressors encountered in medicinal product manufacture and evaluation. The manufacture of therapeutic protein products commonly involves repeat freeze/thaw cycles and lyophilisation that often cause protein denaturation [26], while preclinical and clinical evaluations require exposure to different temperatures. The ideal FGF-2 solution formulation would be stable to lyophilisation, and the lyophilised powder would be sufficiently stable to allow transport and storage without the requirement for refrigeration, and would further confer stability when it is reconstituted into aqueous solutions for administration to patients. Moreover, any excipients applied to stabilise FGF-2 in the formulation should not interfere with the bioactivity of FGF-2.

## 2. Materials and Methods

### 2.1. Materials

Lyophilised, recombinant human FGF-2 (18-kDa isoform, *E. coli* expression system) was kindly provided by Essex Bio-Pharmaceutical Co. (Zhuhai, China). Low viscosity sodium alginate was purchased from Buchi Labortechnik AG (Flawil, Switzerland), sodium chloride, glycine and mannitol were purchased from Ajax Finechem (Tarren Point, Australia), maltodextrin M180 was sourced from the Grain Processing Corporation (Muscatine, IA, USA), D-glucose was purchased from ChemSupply (Gillman, Australia), methylcellulose USP 4000 was purchased from Professional Compounding Chemists of Australia (Matraville, Australia), and human serum albumin, DL-alanine and hydroxypropyl methylcellulose (HPMC) were purchased from Sigma-Aldrich (St. Louis, MO, USA). Deionised water was supplied by a BOSS water system (PSI Water Filters, Launceston, Australia) and used throughout.

### 2.2. Assessment of Stability of FGF-2 as Received from Manufacturer

Lyophilised FGF-2 powder as received from the manufacturer was reconstituted in water at 1 mg/mL (based on dry powder weight), and stored as aliquots (20 to 100 µL) in 0.1 mL Eppendorf^®^ tubes at −20 °C (FJ302V-L, Westinghouse Electric Corporation, Pitsburgh, PA, USA). FGF-2 content was determined by diluting a freshly reconstituted solution on ice to 100 pg/mL (dry powder weight) and analysing immediately using an ELISA kit (Human FGF basic ELISA Kit, Catalog no. KHG0021, Thermo Fisher Scientific, Waltham, MD, USA) as per the supplied protocol [28]. The kit was calibrated with manufacturer-supplied standards, and the calibration curves (*n* = 19) were linear (range: 15.6–1000 pg/mL; *R*^2^ > 0.999) and reproducible, with no significant differences detected in the slope (multiple linear regression analysis; *p* = 0.1136) and intercept (*p* = 0.3119) values.

Frozen FGF-2 solution was thawed at 4 °C (RP372V-R, Westinghouse Electric Corporation) and serially diluted with water to 1.7 ng/mL (as determined by ELISA). Aliquots were added to 0.1 mL Eppendorf^®^ tubes containing water pre-incubated for 2 h at 4° C, 25 °C or 37 °C (Memmert Incubator UF160, In Vitro Technologies, Nobel Park North, Australia) to prepare 50 µL of 315 pg/mL FGF-2 test samples. As this was a preliminary evaluation, the assessment of FGF-2 stability was conducted at a sub-therapeutic concentration (315 pg/mL) in order to conserve FGF-2. The samples (*n* = 4) were incubated at 4 °C, 25 °C or 37 °C for up to 48 h, and their residual FGF-2 content, analysed by ELISA, was expressed as a percent of the baseline FGF-2 content obtained at *t* = 0. Samples were deemed sufficiently stable if their FGF-2 content did not fall by greater than 10%.

### 2.3. Survey of Excipients for Stabilising FGF-2 Solutions

Excipients falling into the 4 protein stabiliser categories of salt, sugar, polymer and protein/amino acids (Table 1) were evaluated for their potential to stabilise FGF-2 aqueous solution. Stored FGF-2 aliquots were thawed at 4 °C, serially diluted with water to 2.5 µg/mL (as confirmed with ELISA), and mixed with an aqueous stock solution of specified excipient. The final excipient concentrations in the test samples are as listed in Table 1, while the final FGF-2 concentration was 770 ng/mL, which corresponds to the label claim of the Beifushu™ eye drops (Essex Bio-Pharmaceutical Co., Zhuhai, China), a commercially available FGF-2 formulation in current clinical use [29]. Control FGF-2 samples were similarly prepared with water alone, i.e., without excipient. Test and control samples (*n* = 3) were stored at 25 °C for 2 h, and their FGF-2 content as determined by ELISA was compared to baseline level (*t* = 0). All samples with an initial FGF-2 concentration of 770 ng/mL were assayed neat as well as diluted 1 in 500 and 1 in 1000 with water, to ensure the residual FGF-2 concentration fell within the range of the standard curve provided by the commercial ELISA kit.

### 2.4. Optimisation of FGF-2 Aqueous Solutions to Processing Stressors

Based on this initial excipient testing, glucose, methylcellulose (MC), human serum albumin (HSA) and alanine were chosen for further studies. FGF-2 aliquots were thawed at 4 °C, serially diluted with water to 2.5 µg/mL (as confirmed by ELISA), and mixed with an aqueous stock solution of the specified excipient(s). Test samples contained final excipient concentrations as specified in Table 2, and FGF-2 concentration of 770 ng/mL. Control FGF-2 samples (no excipient) were prepared by diluting the 2.5 µg/mL FGF-2 solution with water, while control vehicle samples (no FGF-2) were prepared by diluting the stock excipient solution with water. To facilitate description, the vehicles and corresponding FGF-2 samples are identified by specific IDs provided in Table 3.

Test and control samples were incubated for specified periods at 4 °C, 25 °C, and 37 °C, then analysed for FGF-2 content with the ELISA kit. Samples were also exposed to repeated freeze/thaw cycles by incubating the samples at −20 °C for 24 h followed by 30 min at 4 °C before returning the samples to the freezer again, the cycle being repeated a total of 3 times before sample analysis by ELISA. The effects of lyophilisation were studied by freezing the samples at −20 °C for 12 h and lyophilising the frozen samples over 24 h (Alpha 1-2 LDplus, Martin Christ Gefriertrocknungsanlagen GmbH, Osterode am Harz, Germany). The lyophilised samples, stored at −20 °C until analysis, were reconstituted with water to their pre-lyophilisation volume immediately prior to analysis by ELISA. Samples were deemed sufficiently stable if their FGF-2 content did not fall by greater than 10% after processing.

### 2.5. Storage Stability of Lyophilised and Reconstituted FGF-2 Samples

**Figure 1:**
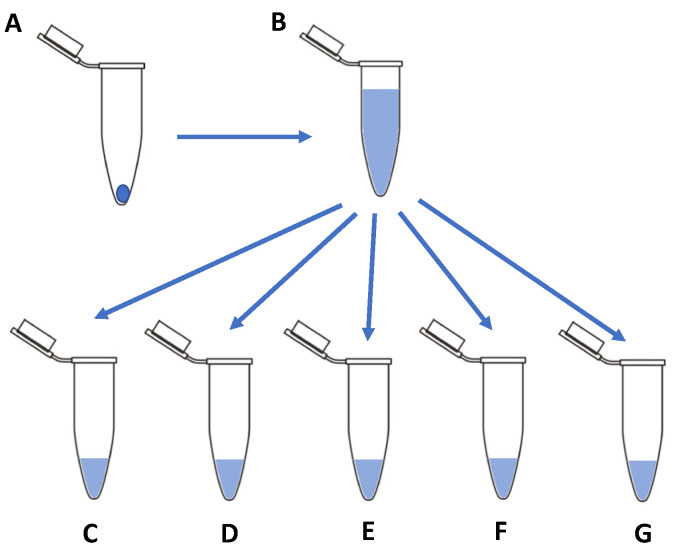
Schematic representation of the methodology used to determine the storage stability of lyophilised and reconstituted FGF-2 solutions. Lyophilised FGF-2 samples (**A**) were stored at −4 °C, 4 °C or 18 °C for up to 12 months. At defined time points, samples were reconstituted with 50 µL of water (**B**). The reconstituted FGF-2 solution was divided into 10-µL aliquots (**C**–**G**) with one aliquot (**C**) immediately analysed for FGF-2 content, and the remaining stored at 4 °C for 24 h (**D**) and 7 days (**E**), and at 18 °C for 24 h (**F**) and 7 days (**G**) before analysis for residual FGF-2 content by ELISA.

F1, F5 and F6 immediately after preparation were measured for baseline FGF-2 content via ELISA (*n* = 3) and lyophilised as 50-µL aliquots in 0.1 mL Eppendorf^®^ tubes. Lyophilised samples were stored at −4° C, 4 °C or 18 °C for up to 12 months, with storage temperatures monitored weekly (SFL−10 to +110, Brannan Thermometers and Gauges, Cleator Moor, UK). At defined storage time points (time 0, 1 week, 2 weeks, 1, 3, 6, 9 and 12 months), triplicate samples were reconstituted with 50 µL of water. The reconstituted solution was processed according to the scheme outlined in Figure 1, with 10 µL removed for baseline FGF-2 content analysis and the remaining solution stored as 10-µL aliquots at 4 °C and 18 °C for 24 h and 7 days before analysis for residual FGF-2 content. Samples were deemed sufficiently stable if the FGF-2 content did not differ from baseline value by greater than 10% after processing.

### 2.6. Data Analysis

Results are expressed as mean ± SD. Data from the preliminary excipient testing study were analysed by one-way ANOVA. All other data were analysed by two-way ANOVA with post-hoc Tukey’s test applied for paired comparison of means, unless stated otherwise. All statistical analyses were completed using GraphPad Prism 8 (San Diego, CA, USA) and a *p* value ≤ 0.05 was considered to be significant.

## 3. Results

### 3.1. Stability of FGF-2 as Received from Manufacturer

**Figure 2:**
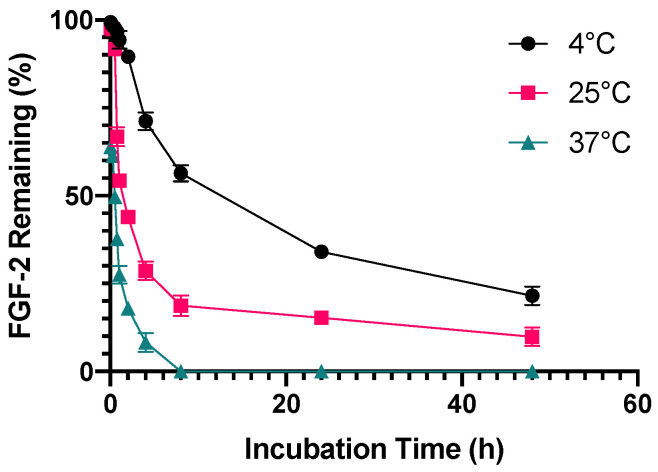
Stability of aqueous FGF-2 solution to different temperatures. FGF-2 solutions (315 pg/mL) reconstituted from the commercial lyophilised powder were incubated at 4 °C, 25 °C and 37 °C for up to 48 h. Residual FGF-2 content measured using an ELISA kit is expressed as a percentage of baseline FGF-2 content (mean ± SD, *n* = 4).

FGF-2 solutions prepared by dissolving the commercial lyophilised powder in water were found to deteriorate very rapidly on storage, even with refrigeration. Samples exposed to 4 °C were stable for only 2 h while those exposed to 25 °C were stable for 30 min. Half-life for the reconstituted FGF-2 solution (315 pg/mL) was 30 min at 37 °C, 1 h at 25 °C and 8 h at 4 °C (Figure 2). As this was a preliminary evaluation, the assessment of FGF-2 stability was conducted at a sub-therapeutic concentration (315 pg/mL) in order to conserve FGF-2. All subsequent studies were completed using a therapeutic dose of FGF-2 (770 ng/mL) to mimic therapeutic applications.

### 3.2. Identification of Potential Stabilisers for Aqueous FGF-2 Solutions

A range of excipients from the classes of salt, sugar, polymer, protein and amino acid (Table 1) were assessed for their ability to confer stability to FGF-2 solutions stored at 25 °C for 2 h. The excipients were employed at concentrations reported to be effective for protein stabilisation [25,30].

**Figure 3:**
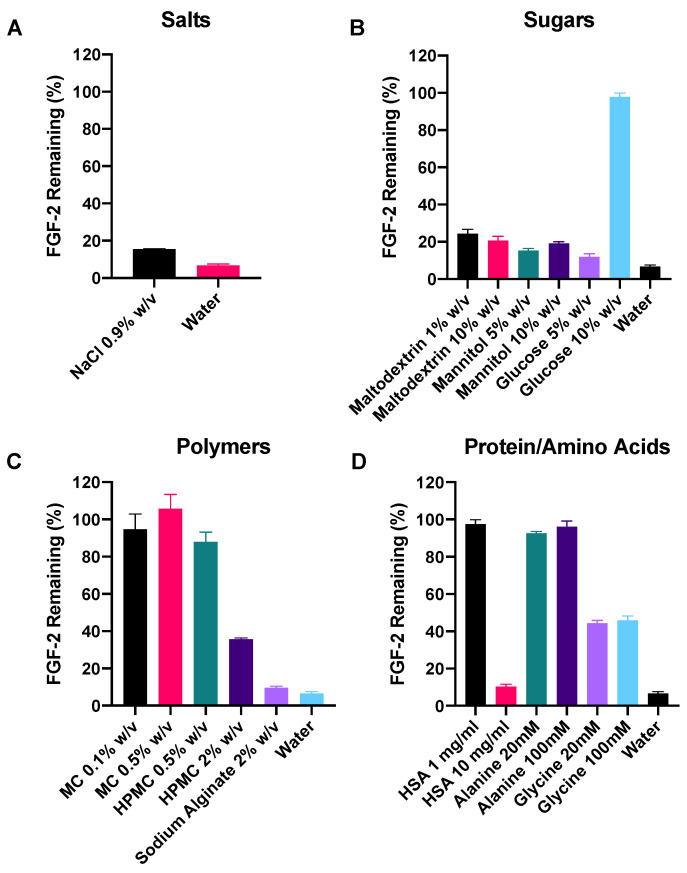
Effect of excipients on the stability of FGF-2 aqueous solution (770 ng/mL) incubated at 25 °C for 2 h. Excipients were selected from the four classes of protein stabilisers, (**A**) salts (**B**) sugars, (**C**) polymers and (**D**) protein/amino acids. FGF-2 content as analysed by ELISA following the incubation period is expressed as a percentage of the baseline FGF-2 content (mean ± SD, *n* = 3). Excipient abbreviations: sodium chloride, NaCl; methylcellulose, MC; hydroxypropyl methylcellulose, HPMC; and human serum albumin, HSA.

Sodium chloride at 0.9% *w/v* was the only salt employed. It significantly reduced FGF-2 degradation after 2 h at 25 °C compared with water alone (*p* < 0.0001, Figure 3A), however, it managed to retain only 15.2% of baseline FGF-2. In the sugar class, only glucose 10% *w/v* was able to successfully stabilise FGF-2 (97.4% of baseline) after 2 h at 25 °C (Figure 3B). Glucose did not produce a sufficient stabilisation effect at 5% *w/v* (11.9% of baseline FGF-2); nor did maltodextrin at 1 and 10% *w/v* (24.3 and 20.5% of baseline FGF-2, respectively) or mannitol at 5 and 10% *w/v* (15.4% and 19.2% of baseline FGF-2, respectively), although all the sugar solutions produced greater stabilisation effects on FGF-2 than water (*p* < 0.0001). Of the polymers, only MC effectively stabilised the FGF-2 for 2 h at 25 °C (Figure 3C), with comparable effectiveness observed for MC at 0.1% *w/v* and 0.5% *w*/*v*. With HPMC 0.5% *w*/*v*, while it retained comparable residual FGF-2 content to MC 0.1% *w/v* (*p* = 0.5806), the mean FGF-2 content in the HMPC 0.5% vehicle was 87.9%, which did not meet the criteria for sufficient FGF-2 stabilisation. HPMC 2% *w/v* and sodium alginate 2% *w/v* also failed to sufficiently stabilise FGF-2 during 2 h incubation at 25 °C. In the protein/amino acids class, HSA 1 mg/mL, alanine 20 mM and alanine 100 mM were able to stabilise FGF-2 at 25 °C for 2 h (Figure 3D). Increasing the HSA concentration to 10 mg/mL did not result in a greater stabilisation effect; instead, the FGF-2 content was comparable to that in the control (FGF-2 in water, *p* = 0.2686). Glycine at 20 mM and 100 mM also failed to sufficiently stabilise the FGF-2 solution over 2 h of exposure at 25 °C.

On the basis of these results, glucose 10% *w*/*v*, MC 0.1 and 0.5% *w*/*v*, HPMC 0.5% *w*/*v*, HSA 1 mg/mL, and alanine 20 and 100 mM were chosen for further evaluation. Although HPMC 0.5% *w/v* did not strictly meet the criteria of an effective FGF-2 stabiliser, it was included due to the mean residual FGF-2 content remaining statistically comparable to the MC solutions.

### 3.3. Stabilisation of FGF-2 Solutions to Temperature Stressors

**Figure 4:**
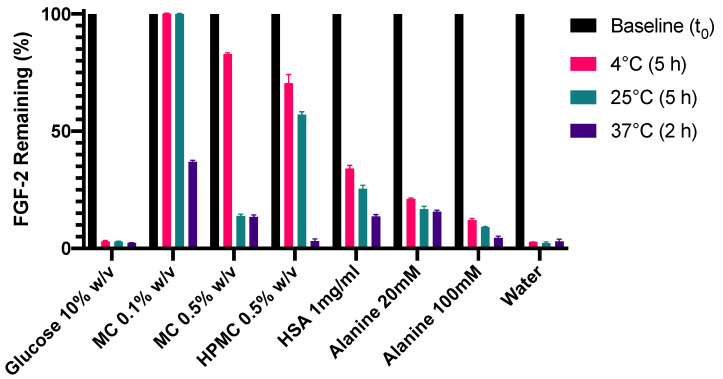
Effect of excipients on the stability of FGF-2 aqueous solutions (770 ng/mL) incubated at 4 °C for 5 h, 25 °C for 5 h and 37 °C for 2 h. Residual FGF-2 content following incubation was measured by ELISA and expressed as a percentage of the baseline concentration (mean ± SD, *n* = 3). Excipient abbreviations: methylcellulose, MC; hydroxypropyl methylcellulose, HPMC; and human serum albumin, HSA.

Stabilisation of FGF-2 solutions to temperature stressors was evaluated at 4 °C, 25 °C and 37 °C. Glucose 10% *w/v* was not effective at sufficiently stabilising the FGF-2 solution at all three prescribed storage conditions whereas MC 0.1% *w/v* showed a strong stabilisation effect (Figure 4), retaining 100% FGF-2 content after 5 h at 4 °C and 5 h at 25 °C. MC 0.1% *w/v* also produced superior FGF-2 stabilisation at 37 °C compared to the other excipients (*p* < 0.0001), but was not successful at maintaining FGF-2 content above 90% after 2 h at 37 °C. The effectiveness of the polymers in stabilising the FGF-2 solution to the prescribed temperature stressors may be ranked in the decreasing order of MC 0.1% *w/v* > HPMC 0.5% *w/v* > MC 0.5% *w*/*w*. In the protein/amino class, HSA 1 mg/mL was more effective than alanine 20 mM, and alanine in turn was more effective at 20 mM than at 100 mM in stabilising FGF-2 at all three storage conditions (*p* < 0.0001). None of the excipients were able to sufficiently stabilise the FGF-2 solution for 2 h at 37 °C, although MC 0.5% *w*/*v*, HSA 1 mg/mL and alanine 20 mM produced a greater stabilisation effect when compared to water alone (*p* < 0.0001).

**Figure 5:**
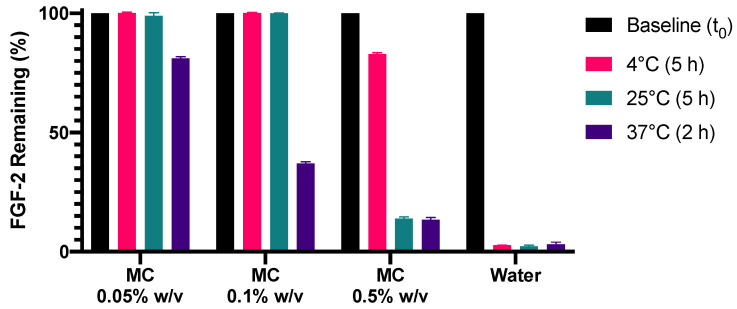
Effect of methylcellulose (MC) concentration on the stability of aqueous FGF-2 solutions (770 ng/mL) incubated at 4 °C for 5 h, 25 °C for 5 h and 37 °C for 2 h. Residual FGF-2 contents following the prescribed incubation period were measured by ELISA and expressed as a percentage of the baseline concentration (mean ± SD, *n* = 3).

With MC exhibiting concentration-dependent stabilisation effects on FGF-2, being more effective at 0.1% than at 0.5% (Figure 4), the stabilisation effect of MC was evaluated at a lower concentration of 0.05% *w*/*v*. Figure 5 shows that MC at 0.05% *w/v* indeed resulted in improved stability of FGF-2 at 37 °C (*p* < 0.0001). Compared with MC 0.1% and MC 0.5%, which retained residual FGF-2 contents of 36.9% and 13.4%, respectively, after 2 h at 37 °C, MC 0.05% could retain 81% of baseline FGF-2 content after similar heat exposure.

As no single excipient was able to sufficiently stabilise FGF-2 against thermal degradation at 37 °C, combinations of the excipients were applied to evaluate for synergistic effects [31,32,33]. Combinations of MC 0.05% *w/v* with either alanine 20 mM, HSA 1 mg/mL or both alanine 20 mM and HSA 1 mg/mL, were evaluated. To facilitate discussion, the blank vehicles and corresponding FGF-2 formulations will henceforth be identified by their respective ID as listed in Table 3.

**Figure 6:**
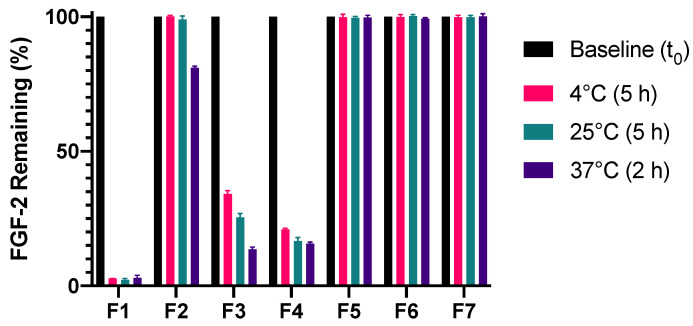
Effect of excipient combinations on the stability of FGF-2 aqueous solutions (770 ng/mL) incubated at 4 °C for 5 h, 25 °C for 5 h and 37 °C for 2 h. Residual FGF-2 content following incubation was measured by ELISA and expressed as a percentage of the baseline concentration (mean ± SD, *n* = 3). Abbreviations of samples: F1, FGF-2 in water; F2, FGF-2 and 0.05% *w/v* methylcellulose (MC) in water; F5, FGF-2, 0.05% *w/v* MC and 20 mM alanine in water; F6, FGF-2, 0.05% *w/v* MC and 1 mg/mL human serum albumin (HSA) in water; and F7, FGF-2, 0.05% *w/v* MC, 20 mM alanine and 1 mg/mL HSA in water.

**Figure 7:**
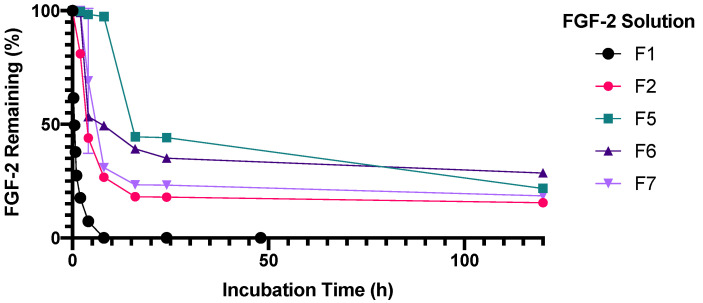
Effect of excipient combinations on the stability of FGF-2 aqueous solutions (770 ng/mL) incubated at 37 °C for up to 5 days. Residual FGF-2 content following incubation was measured by ELISA and expressed as a percentage of the baseline concentration (mean ± SD, *n* = 3). Abbreviation of samples: F1, FGF-2 in water; F2, FGF-2 and 0.05% *w/v* methylcellulose (MC) in water; F5, FGF-2, 0.05% *w/v* MC and 20 mM alanine in water; F6, FGF-2, 0.05% *w/v* MC and 1 mg/mL human serum albumin (HSA) in water; and F7, FGF-2, 0.05% *w/v* MC, 20 mM alanine and 1 mg/mL HSA in water.

Unlike F2 (MC alone), F3 (alanine alone) and F4 (HSA alone), there were insignificant losses of FGF-2 content in F5, F6 and F7 following incubation for 5 h at 4 °C, 5 h at 25 °C and 2 h at 37 °C (Figure 6). This indicates that FGF-2 was effectively stabilised against the prescribed storage conditions when prepared with the combinations of MC with alanine (F5), MC with HSA (F6) or MC with alanine and HSA (F7). F5 was also stable when incubation at 37 °C was extended to 8 h (97% residual FGF-2 content) (Figure 7), but the stabilisation effect was not sustained on further prolongation of incubation. By comparison, F6 and F7 showed significant deterioration on prolonged incubation at 37 °C, retaining less than 50% of baseline FGF-2 content at 8 h of incubation at 37 °C.

### 3.4. Stabilisation of FGF-2 Solutions to Repeat Freeze/Thaw and Lyophilisation Stressors

**Figure 8:**
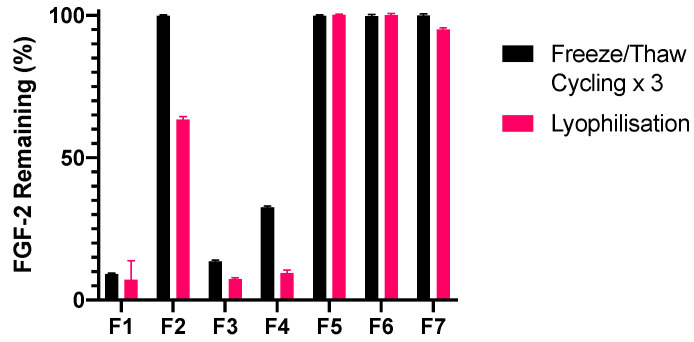
Effect of excipients on the stability of FGF-2 aqueous solutions (770 ng/mL) exposed to processing stressors. Solutions were subject to three freeze/thaw cycles or lyophilisation, and the residual FGF-2 content was measured by ELISA and expressed as a percentage of the baseline concentration (mean ± SD, *n* = 3). Sample abbreviations: F1, FGF-2 in water; F2, FGF-2 and 0.05% *w/v* methylcellulose (MC) in water; F3, FGF-2 and 20 mM alanine in water; F4, FGF-2 and human serum albumin (HSA) in water; F5, FGF-2, 0.05% *w/v* MC and 20 mM alanine in water; F6, FGF-2, 0.05% *w/v* MC and 1 mg/mL HSA in water; and F7, FGF-2, 0.05% *w/v* MC, 20 mM alanine and 1 mg/mL HSA in water.

F2, F5, F6 and F7 were stable against repeated freeze/thaw cycles, with ≥99.8% baseline FGF-2 measured in these samples after the third cycle of processing (Figure 8). By comparison, F3 and F4 were unable to sufficiently protect FGF-2 during repeated freeze/thaw cycles, with F3 no better than the F1 control (*p* = 0.0929) and F4, while more stable than F1 (*p* < 0.0001), demonstrating a residual FGF-2 content of 32.5% which was unacceptably low.

There was substantial loss of FGF-2 content following lyophilisation of F2, F3 and F4, all of which contain a single stabiliser excipient (Figure 8). By contrast, the three solutions containing excipient combinations were deemed stable to lyophilisation, with F7 being slightly, though significantly, less stable than F5 and F6 (*p* = 0.0410). The collective data suggest that having the three excipients in combination (F7) was no more effective in stabilising the FGF-2 solution than the dual combinations of MC with alanine (F5) or MC with HSA (F6). Thus, only F5 and F6 were progressed to further storage stability studies.

### 3.5. Storage Stability of Lyophilised and Reconstituted FGF-2 Samples

F5 and F6 were lyophilised and evaluated for storage stability as lyophilised powders and as reconstituted solutions prepared by adding water to the lyophilised powders. F1 (water alone) served as the control. The lyophilised FGF-2 powders were stored at −4 °C, 4 °C and 18 °C for specified periods of up to 12 months, and were deemed sufficiently stable if the FGF-2 content, as measured with ELISA following reconstitution of the powders with water, did not differ by greater than 10% from baseline FGF-2 content measured in the pre-lyophilised solutions. The mean baseline FGF-2 contents for F1, F5 and F6 were comparable (*p* = 0.2503), and were 768, 769 and 771 ng/mL, respectively.

**Figure 9:**
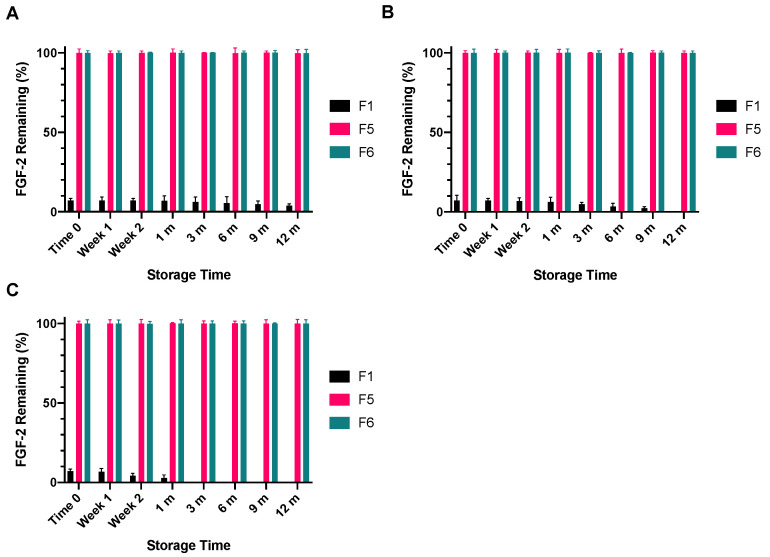
Stability of FGF-2 aqueous solutions (770 ng/mL) upon lyophilisation and storage. FGF-2 in solutions F1 (water alone), F5 (MC 0.05% *w/v* and alanine 20 mM) and F6 (MC 0.05% *w/v* and HSA 1 mg/mL) were lyophilised over 24 h then stored at −4 °C (**A**), 4 °C (**B**) or 18 °C (**C**) for up to 12 months. At defined storage time points, the lyophilised powders were reconstituted with water and the FGF-2 content as determined by ELISA is expressed as a percentage of the baseline FGF-2 content determined in the FGF-2 solutions prior to lyophilisation (mean ± SD, *n* = 3).

Immediately following lyophilisation, only 7.1% of the baseline FGF-2 content of F1 had remained, representing a significant drop in FGF-2 content (Figure 9). This FGF-2 level was maintained when the lyophilised F1 powder was stored at −4 °C for 12 months (*p* = 0.9367). When the storage temperature was increased to 4 °C, the FGF-2 content in F1 was maintained for only 9 months, with further storage leading to undetectable levels of FGF-2 in the lyophilised F1 powder at 12 months. Storage at the higher temperature of 18 °C caused the FGF-2 content of the lyophilised F1 powder to fall below the detectable limit at 3 months. By comparison, F5 and F6 were not only stable to lyophilisation, but the lyophilised F5 and F6 powders were also stable to storage at −4 °C, 4 °C and 18 °C for up to 12 months. The FGF-2 contents in the lyophilised F5 and F6 powders remained above 99% of baseline levels throughout the duration of the prescribed storage conditions.

**Figure 10:**
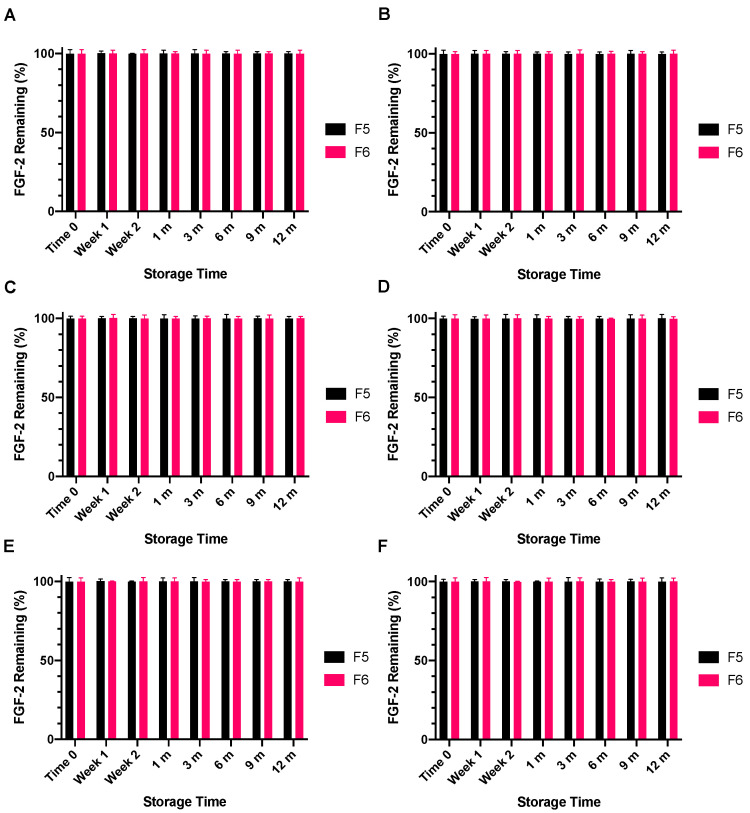
Effect of excipients on the stability of reconstituted FGF-2 aqueous solutions (770 ng/mL) over a 24-h period. FGF-2 in solutions F5 (MC 0.05% *w/v* and alanine 20 mM) and F6 (MC 0.05% *w/v* and human serum albumin 1 mg/mL) were lyophilised over 24 h and the dry powders stored at −4 °C (**A**,**B**), 4 °C (**C**,**D**) or 18 °C (**E**,**F**) for up to 12 months. At defined storage time points, the FGF-2 powders were reconstituted with water, and the resultant solutions were stored for 24 h at 4 °C (**A**,**C**,**E**) or 24 h at 18 °C (**B**,**D**,**F**). The FGF-2 content in the stored solutions was determined by ELISA and expressed as a percentage of the baseline FGF-2 content determined in the FGF-2 solutions prior to lyophilisation (mean ± SD, *n* = 3).

**Figure 11:**
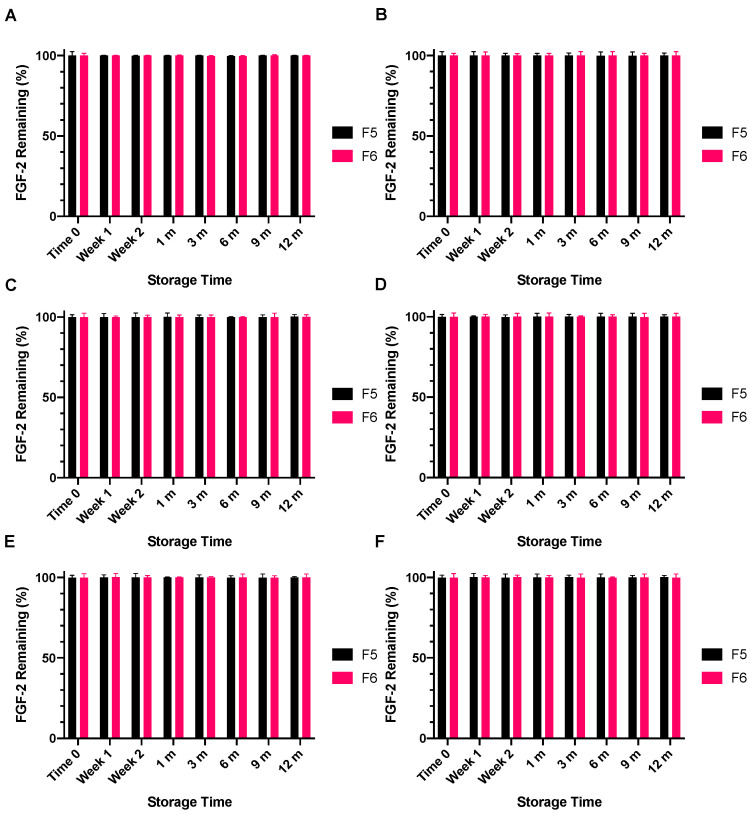
Effect of excipients on the stability of reconstituted FGF-2 aqueous solutions (770 ng/mL) over a 7-day period. FGF-2 in solutions F5 (MC 0.05% *w/v* and alanine 20 mM) and F6 (MC 0.05% *w/v* and human serum albumin 1 mg/mL) were lyophilised over 24 h and the dry powders stored at −4 °C (**A**,**B**), 4 °C (**C**,**D**) or 18 °C (**E**,**F**) for up to 12 months. At the defined storage time points, the FGF-2 powders were reconstituted with water, and the resultant solutions were stored for 7 days at 4 °C (**A**,**C**,**E**) or 7 days at 18 °C (**B**,**D**,**F**). The FGF-2 content in the stored solutions was determined by ELISA and expressed as a percentage of the baseline FGF-2 content determined in the FGF-2 solutions prior to lyophilisation (mean ± SD, *n* = 3).

The lyophilised F1, F5 and F6 powders following storage at −4 °C, 4 °C or 18 °C for specified time points were reconstituted with water and the resultant solutions were stored at either 4 °C or 18 °C for 24 h or 7 days. Solutions prepared by reconstituting the lyophilised F1 powder were not stable to storage at 4 °C or 18 °C for 24 h, the FGF-2 content in all F1 reconstituted solutions falling below the detectible limit of the ELISA assay (data not presented). By comparison, the solutions reconstituted from the lyophilised F5 and F6 remained stable for not only 24 h (Figure 10) but also 7 days (Figure 11) at both storage temperatures, retaining >99% of the baseline FGF-2 content throughout the prescribed storage conditions.

## 4. Discussion

Data from this study highlight the need for effective stabilisation strategies to allow FGF-2 to be formulated into viable, safe and effective medicinal products. The commercial lyophilised FGF-2 used in this study was recommended by the manufacturer to be reconstituted with water, however, the FGF-2 reconstituted in water alone (F1) underwent a rapid loss of FGF-2 content, as detected by ELISA, at ambient and refrigerated temperatures, indicating a loss of functionality. Our findings are congruent with published data, which have shown FGF-2 solutions to lose 50% functionality after just 46 min at 25 °C, and the FGF-2 functional half-life to decrease to 37 min when the storage temperature was increased to 37 °C [34,35]. Interestingly, as FGF-2 concentration increased from 315 pg/mL, in the preliminary assessment of FGF-2 stability, to 770 ng/mL, in subsequent studies, it was observed that a higher FGF-2 content corresponded with a greater rate of FGF-2 loss. This is potentially due to the formation of FGF-2 multimers, rendering the FGF-2 undetectable by ELISA. This self-association of FGF-2 has been previously reported to occur in a concentration dependent manner, which may explain why FGF-2 stability in water decreased as the concentration increased [36]. In order to fully characterise this effect, it could be useful to determine the stability of FGF-2 solutions of varying concentrations.

Although no accepted limits have been set for the stability of biological products in any of the current regulatory guidelines, with stability limits instead determined on a molecule-by-molecule basis, this study adopted a cut-off of 90% FGF-2 content remaining as being indicative of sufficient FGF-2 stability for therapeutic use. This cut-off was chosen on the basis of the high quality assurance standards required for pharmaceutical products [37], as well as the economic implications of FGF-2 loss in the context of commercial manufacturing. To render the FGF-2 solution sufficiently stable for processing into acceptable medicinal products that could be transported and stored without requiring −20 °C storage, this study explored a range of excipients which were expected to act via different mechanisms. Salts can stabilise protein via nonspecific ionic effects [38,39,40], as well as via specific effects that are dependent on salt type [41,42,43], solution surface tension [44], and preferential interaction between salt and protein [42,43]. NaCl at concentrations >9.9% *w/v* has been shown to negate the stabilising effects of heparin on FGF-2 by disrupting the binding of heparin to FGF-2 [17]. However, NaCl as a pharmaceutical excipient is more commonly employed at or below the isotonic concentration of 0.9% *w*/*v*, and this concentration was employed for the stability study. NaCl at 0.9% *w/v* did not stabilise the FGF-2 solution against thermal degradation, nor did it destabilise FGF-2 compared with the F1 control. In contrast, human prior protein had reduced stability in the presence of low NaCl concentrations, possibly due to the ion-induced destabilisation of salt bridges within the protein [45]. The FGF-2 structure does not contain salt bridges [46], perhaps accounting for the lack of effect of low concentrations of NaCl on its stability.

Sugars are well known protein stabilisers, and are widely employed as cryoprotectants. Sugars increase the surface tension and viscosity of solutions to protect proteins against aggregation [25]. They also lead to preferential hydration of proteins, with the resultant reduction in protein free energy providing a potential secondary mechanism for protein stabilisation in aqueous vehicles [47]. These effects are sugar-specific, e.g., mannitol did not protect FGF-2 against thermal degradation in this study although it was successful at stabilising FGF-7 (keratinocyte growth factor) in another study [26]. Glucose in this study was effective at protecting FGF-2 against thermal degradation in a concentration-dependent manner, which correlates with its anticipated mechanism of action, as glucose 10% *w/v* would have higher solution viscosity and thus will be better able to minimise protein aggregation than glucose 5% *w*/*v*.

Amino acids with no net charge, such as alanine and glycine, are also common cryoprotectants of protein formulations, providing stability through weak electrostatic interactions [25]. In this study, alanine, but not glycine, met the criteria of an effective stabiliser for FGF-2. Alanine has a methyl side chain that provides greater stearic hindrance, which might account for its greater capacity to stabilise FGF-2 than the simpler glycine molecule [48]. At higher temperatures and longer incubation, alanine at the lower concentration of 20 mM was significantly more effective at stabilising FGF-2 than at the higher concentration of 100 mM. Alanine is a zwitterion in solutions of near neutral pH, and has greater tendency towards self-aggregation at higher concentration that might have resulted in fewer alanine molecules for electrostatic interactions with the FGF-2 at 100 mM than at 20 mM [49].

Proteins like HSA and bovine serum albumin are able to stabilise the structure of other proteins through ionic, electrostatic and hydrophobic interactions [26,27], and they are used as excipients of commercial lyophilised FGF-2 products; an example is the recombinant human basic fibroblast growth factor (CAS Number 106096-93-9, Merck, Bayswater, Australia). In this study, HSA at 1 mg/mL effectively stabilised FGF-2 solutions at 25 °C for 2 h; however, increasing the HSA concentration to 10 mg/mL negated the stabilisation effect, possibly because, as a protein, HSA is also prone to self-aggregation at higher concentrations.

The polymers investigated in this study, MC, HPMC and sodium alginate, are all hydrogels [50,51,52] potentially capable of increasing the molecular density and viscosity of aqueous solutions to prevent protein aggregation [25]. This effect appears to be favourable at low polymer concentrations for MC and HPMC, probably because a micro-heterogeneous two-phase system can develop at higher polymer concentrations that then promote protein aggregation in the phase at which it is present at higher concentration [25,53]. Unlike MC and HPMC, sodium alginate did not produce a significant stabilisation effect on FGF-2 solution. This variation in stabilisation effect among the polymers, which was observed with excipients across the different classes and also with different concentrations of the same excipient, simply confirms the highly individualised approach that is required to achieve effective stabilisation of aqueous FGF-2 solutions [26]. Based on these results, it is possible that excipients may also demonstrate equal or greater stabilisation potential at concentrations other than those which have been explored in this study. Unfortunately, due to the limited quantity of FGF-2 available, further exploration was not possible; however, this could be addressed in the future, using a design of experiments protocol to determine the upper and lower excipient concentration limits for the effective stabilisation of FGF-2.

Lyophilisation is a common technique for prolonging the shelf life of commercial protein products which are insufficiently stable to storage as aqueous solutions [54]. The processes associated with lyophilisation does, however, result in protein degradation, and a cryoprotectant that minimises structural damage, often by forming hydrogen bonds with the protein as water molecules are displaced during the lyophilisation process, is required to stabilise the protein [26]. Commercial FGF-2 lyophilised powders often contain a cryoprotectant, however, the lyophilised FGF-2 powder employed in this study did not contain additives (as per manufacturer’s advice). It is therefore not surprising that the F1 aqueous solutions reconstituted with the commercial FGF powder displayed a 93% loss in detectable FGF-2 following processing by lyophilisation.

Lyophilisation involves two major stressors, freezing and drying, with both processes capable of damaging a protein structure. Many studies have shown that a stabiliser effective at protecting a protein against freezing may not be effective at stabilising the protein against the process of lyophilisation [26]. This was certainly the case with MC; although MC could stabilise FGF-2 against multiple freeze-thaw cycles, it was ineffective at preserving FGF-2 against lyophilisation when applied alone. Effective stabilisation of FGF-2 against lyophilisation required MC to be combined with alanine and/or HSA. Such synergism in stabilisation effects between different classes of protein stabilisers is not unusual [55,56]. For the FGF-2 aqueous solutions, there is clear advantage in combining the stabilisation effects of MC with those of alanine and/or HSA, to protect FGF-2 against lyophilisation and the subsequent storage and reconstitution of the lyophilised protein powder. The successful stabilisation of a protein against lyophilisation does not always correlate with a capacity to provide storage stability. Elastase, lyophilised without any excipients, was found to have retained its baseline activity immediately following lyophilisation; however, 70% of its activity was lost over the subsequent 2-week storage period [57].

Long term stability of the FGF-2 solutions was conducted in an incubator set at 18 °C in this study as the ambient temperature in the laboratory fluctuated widely over the course of the study. A temperature of 20–25 °C is more widely accepted as representative of ambient temperature, and future stability studies may be conducted at 25 °C for the stabilised FGF-2 solutions. However, as there was no significant difference in stability following storage of the stabilised FGF-2 solutions for 5 h at 4 °C and 5 h at 25 °C (Figure 6), there is unlikely to be a difference in stability based on storage at 18 °C and 25 °C at least in the short term.

The focus of this study was the stabilisation of FGF-2 for direct therapeutic use as a medicinal product, and to enable FGF-2 processing into more complex medicinal products. It is recognised that this research also has implications for cell culture and other basic research applications where FGF-2 stability is of concern. However, as the conditions related to these applications, including different applied FGF-2 concentrations and media, were not assessed in this study, further research would be required. Nonetheless, the stabilised FGF-2 solutions and lyophilised powders identified in this study may provide a foundation for further investigation.

The quantification of FGF-2 is integral to this stability study. FGF-2 has been detected by high performance liquid chromatography (HPLC) and Western Blot [17,58,59,60]. Western Blot relies on denaturation of the protein to allow its movement through the gel and is unable to determine the proportion of total protein retaining the correct tertiary structure. HPLC performed using a heparin-affinity column allows FGF-2 in the correct tertiary structure to be detected, however this technique may not be sensitive for the quantification of low FGF-2 concentrations [59]. In order to quantify the biologically relevant concentrations (pg–µg/mL) of FGF-2 applied in this study, the ELISA assay was applied. The ELISA assay is highly sensitive and specific as it requires FGF-2 to retain the correct tertiary structure for antibody binding and can quantify low levels of FGF-2 in the active conformation, which is indicative of protein functionality.

Although the ELISA results for stabilised FGF-2 aqueous solutions presented in this study appear indicative of a greater retention of FGF-2 functionality than control, the in vitro efficacy of these formulations must be confirmed prior to the clinical translation of this research. This research is the subject of a follow-up study evaluating the in vitro efficacy of stabilised FGF-2 solutions for wound healing applications (manuscript submitted to *Pharmaceutics* October 2021).

## 5. Conclusions

This study has clearly demonstrated the advantage of combining excipients to stabilise FGF-2 against thermal and processing stressors encountered in medicinal product manufacture and testing, and further provided data on long term storage stability. Although the combination of MC, alanine and HSA (F7) had performed well in the stability studies, the data suggested no advantage to using a combination of all three stabilisers. Considering it is desirable from a toxicity and cost perspective to use the least number and lowest concentrations of excipients to achieve a specified outcome for a medicinal product, the simpler F5 (FGF-2, 0.05% *w/v* MC and 20 mM alanine in water) and F6 (FGF-2, 0.05% *w/v* MC and 1 mg/mL HSA in water) formulations would be preferred. F5 and F6 are viable formulations of FGF-2 that are applicable as medicinal solutions, or for further processing into more sophisticated medicinal products as they are stable to a range of temperatures, freeze/thaw cycles, lyophilisation, and storage as lyophilised powders and aqueous solutions reconstituted from the lyophilised powders.

## 6. Patents

This work is the subject of Patent Application AU 2021902450.

## Figures and Tables

**Table 1:** Excipients applied to assess for their potential to stabilise FGF-2 aqueous solutions.

Excipient	Excipient Concentration
Sodium chloride (NaCl)	0.9% *w*/*v*
Maltodextrin	1% *w*/*v*; 10% *w*/*v*
Mannitol	5% *w*/*v*; 10% *w*/*v*
Glucose	5% *w*/*v*; 10% *w*/*v*
Methylcellulose (MC)	0.1% *w*/*v*; 0.5% *w*/*v*
Hydroxypropyl methylcellulose (HPMC)	0.5% *w*/*v*; 2% *w*/*v*
Sodium alginate	2% *w*/*v*
Human serum albumin (HSA)	1 mg/mL; 10 mg/mL
Alanine	20 mM; 100 mM
Glycine	20 mM; 100 mM

**Table 2:** Composition of potential FGF-2 stabilisation vehicles.

Excipient(s)	Final Excipient Concentration(s)
Glucose	10% *w*/*v*
Methylcellulose (MC)	0.05% *w*/*v*; 0.10% *w*/*v*; 0.50% *w*/*v*
Human Serum Albumin (HSA)	1 mg/mL
Alanine	20 mM
MC and alanine	MC 0.05% *w/v* and alanine 20 mM
MC and HSA	MC 0.05% *w/v* and HSA 1 mg/mL
MC and HSA and alanine	MC 0.05% *w*/*v*, HSA 1 mg/mL and alanine 20 mM

**Table 3:** Identification key for blank vehicles and corresponding FGF-2 solutions (770 ng/mL) prepared using the vehicle.

Vehicle ID	Composition of Aqueous Vehicle	Corresponding FGF-2 Sample ID
1	Water (control)	F1
2	Methylcellulose (MC; 0.05% *w*/*v*)	F2
3	Alanine (20 mM)	F3
4	Human serum albumin (HSA; 1 mg/mL)	F4
5	MC (0.05% *w*/*v*) and alanine (20 mM)	F5
6	MC (0.05% *w*/*v*) and HSA (1 mg/mL)	F6
7	MC (0.05% *w*/*v*), alanine (20 mM) and HSA (1 mg/mL)	F7

## Data Availability

The data presented in this study are available in “Stabilisation of Recombinant Human Basic Fibroblast Growth Factor (FGF-2) Against Stressors Encountered in Medicinal Product Processing and Evaluation”.
